# Genomic diversity of *Escherichia coli* isolates from non-human primates in the Gambia

**DOI:** 10.1099/mgen.0.000428

**Published:** 2020-09-14

**Authors:** Ebenezer Foster-Nyarko, Nabil-Fareed Alikhan, Anuradha Ravi, Gaëtan Thilliez, Nicholas M. Thomson, David Baker, Gemma Kay, Jennifer D. Cramer, Justin O’Grady, Martin Antonio, Mark J. Pallen

**Affiliations:** ^1^​ Quadram Institute Bioscience, Norwich Research Park, Norwich, Norfolk, UK; ^2^​ Medical Research Council Unit the Gambia at the London School of Hygiene and Tropical Medicine, Atlantic Boulevard Road, Fajara, Gambia; ^3^​ American Public University System, Charles Town, WV, USA; ^4^​ Microbiology and Infection Unit, Warwick Medical School, University of Warwick, Coventry, UK; ^5^​ School of Veterinary Medicine, University of Surrey, Guildford, Surrey, UK

**Keywords:** Non-human primates, *Escherichia coli*, phylogenomic diversity, Extraintestinal pathogenic *E. coli*

## Abstract

Increasing contact between humans and non-human primates provides an opportunity for the transfer of potential pathogens or antimicrobial resistance between host species. We have investigated genomic diversity and antimicrobial resistance in *Escherichia coli* isolates from four species of non-human primates in the Gambia: *Papio papio* (*n*=22), *Chlorocebus sabaeus* (*n*=14), *Piliocolobus badius* (*n*=6) and *Erythrocebus patas* (*n*=1). We performed Illumina whole-genome sequencing on 101 isolates from 43 stools, followed by nanopore long-read sequencing on 11 isolates. We identified 43 sequence types (STs) by the Achtman scheme (ten of which are novel), spanning five of the eight known phylogroups of *E. coli *. The majority of simian isolates belong to phylogroup B2 – characterized by strains that cause human extraintestinal infections – and encode factors associated with extraintestinal disease. A subset of the B2 strains (ST73, ST681 and ST127) carry the *pks* genomic island, which encodes colibactin, a genotoxin associated with colorectal cancer. We found little antimicrobial resistance and only one example of multi-drug resistance among the simian isolates. Hierarchical clustering showed that simian isolates from ST442 and ST349 are closely related to isolates recovered from human clinical cases (differences in 50 and 7 alleles, respectively), suggesting recent exchange between the two host species. Conversely, simian isolates from ST73, ST681 and ST127 were distinct from human isolates, while five simian isolates belong to unique core-genome ST complexes – indicating novel diversity specific to the primate niche. Our results are of planetary health importance, considering the increasing contact between humans and wild non-human primates.

## Data Summary

The raw sequences and polished assemblies from this study are available in the National Centre for Biotechnology Information (NCBI) Short Read Archive, under the BioProject accession number PRJNA604701. The full list and characteristics of these strains and other reference strains used in the analyses are presented in Table 1 and Files S2, S4–S8 (available in the online version of this article).

**Impact Statement**

Little is known about the population structure, virulence potential and the burden of antimicrobial resistance among *Escherichia coli* from wild non-human primates, despite increased exposure to humans through the fragmentation of natural habitats. Previous studies, primarily involving captive animals, have highlighted the potential for bacterial exchange between non-human primates and humans living nearby, including strains associated with intestinal pathology. Using multiple-colony sampling and whole-genome sequencing, we investigated the strain distribution and population structure of *E. coli* from wild non-human primates from the Gambia. Our results indicate that these monkeys harbour strains that can cause extraintestinal infections in humans. We document the transmission of virulent *E. coli* strains between monkeys of the same species sharing a common habitat and evidence of recent interaction between strains from humans and wild non-human primates. Also, we present complete genome assemblies for five novel sequence types of *E. coli *.

## Introduction

*Escherichia coli* is a highly versatile species, capable of adapting to a wide range of ecological niches and colonizing a diverse range of hosts [1, 2]. In humans, *E. coli* colonizes the gastrointestinal tract as a commensal, as well as causing intestinal and extraintestinal infection [2]. *E. coli* is also capable of colonizing the gut in non-human primates [3], where data from captive animals suggest that gut isolates are dominated by phylogroups B1 and A, which, in humans, encompass commensals as well as strains associated with intestinal pathology [4, 5]. *E. coli* strains encoding colibactin, or cytotoxic necrotizing factor 1 have been isolated from healthy laboratory rhesus macaques [6], while enteropathogenic *E. coli* strains can – in the laboratory – cause colitis in marmosets [7], rhesus macaques infected with simian immunodeficiency virus [8] and cotton-top tamarins [9].

There are two potential explanations for the occurrence of *E. coli* in humans and non-human primates. Some bacterial lineages may have been passed on through vertical transmission within the same host species for long periods, perhaps even arising from ancestral bacteria that colonized the guts of the most recent common ancestors of humans and non-human primate species [10, 11]. In such a scenario, isolates from non-human primates would be expected to be novel and distinct from the diversity seen in humans [11]. However, there is also clearly potential for horizontal transfer of strains from one host species to another [12].

The exchange of bacteria between humans and human-habituated animals, particularly non-human primates, is of interest in light of the fragmentation of natural habitats globally [13–15]. We have seen that wild non-human primates in the Gambia are frequently exposed to humans through tourism, deforestation and urbanization [16]. In Uganda, PCR-based studies have suggested transmission of *E. coli* between humans, non-human primates and livestock [17, 18]. These studies are complicated by the low resolution of PCR-based methods; nonetheless, their findings highlight the possibility that wild non-human primates may constitute a reservoir for the zoonotic spread of *E. coli* strains associated with virulence and antimicrobial resistance to humans. Alternatively, humans might provide a reservoir of strains with the potential for anthroponotic spread to animals – or transmission might occur in both directions [19].

We do not know how many different lineages can co-exist within the same non-human primate host. Such information may help us contextualize the potential risks associated with transmission of bacterial strains between humans and non-human primates. In humans, up to 11 serotypes could be sampled from picking colonies from individual stool samples [20–22].

To address these issues, we have exploited whole-genome sequencing to explore the population structure and phylogenomic diversity of *E. coli* in wild non-human primates from rural and urban Gambia.

## Methods

### Study population and sample collection

**Fig. 1.**
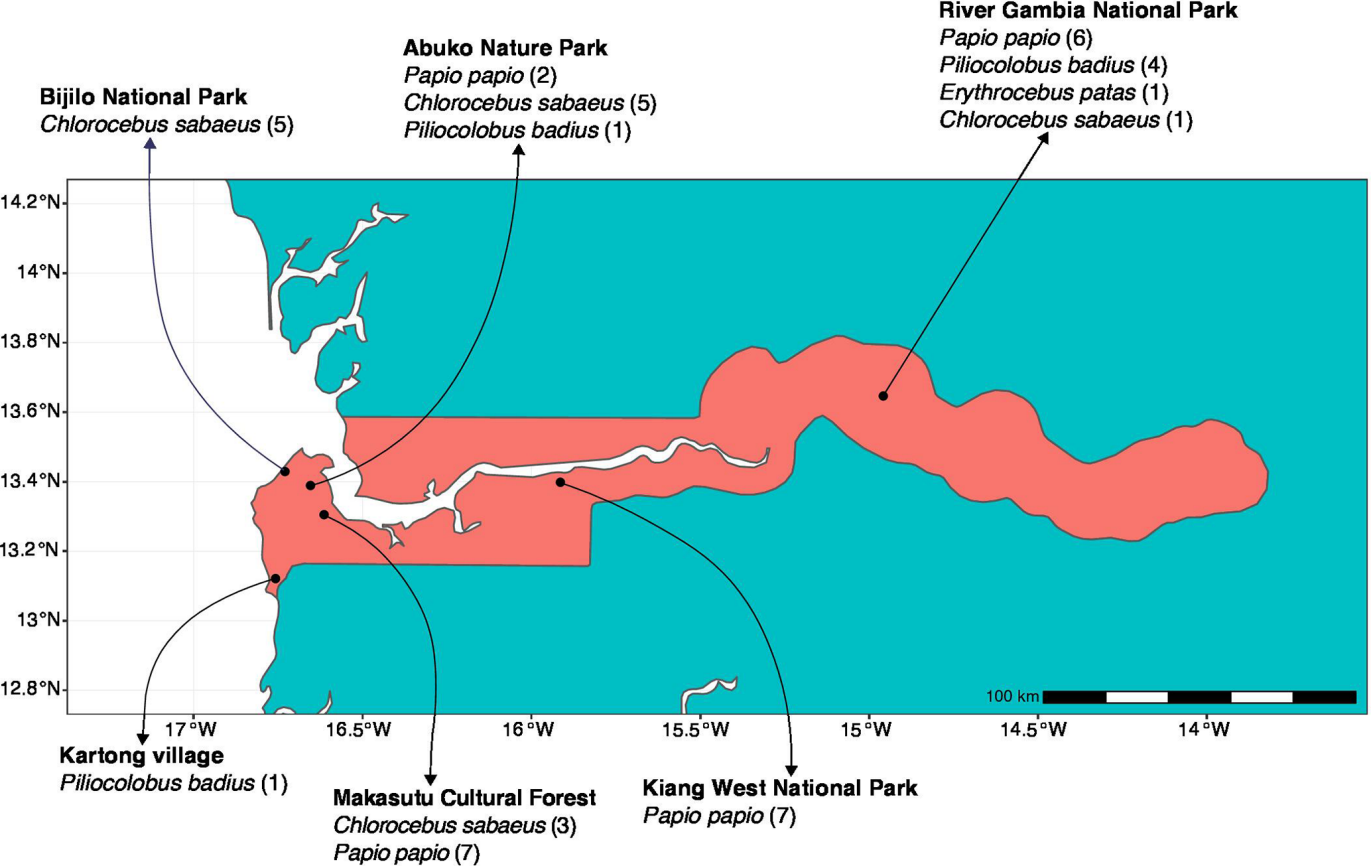
Study sites and distribution of study subjects.

In June 2017, wild non-human primates were sampled from six sampling sites in the Gambia: Abuko Nature Reserve (riparian forest), Bijilo Forest Park (coastal fenced woodland), Kartong village (mangrove swamp), Kiang West National park (dry-broad-leaf forest), Makasutu Cultural Forest (ecotourism woodland) and River Gambia National park (riparian forest) (Fig. 1). The sampling was opportunistic and throughout the range of the primates in the country (all four of the diurnal non-human primate species indigenous to the Gambia), where primates overlap with human communities to varying degrees. Monkeys in Abuko and Bijilo are frequently hand-fed by visiting tourists, despite guidelines prohibiting this practice [16].

**Fig. 2.**
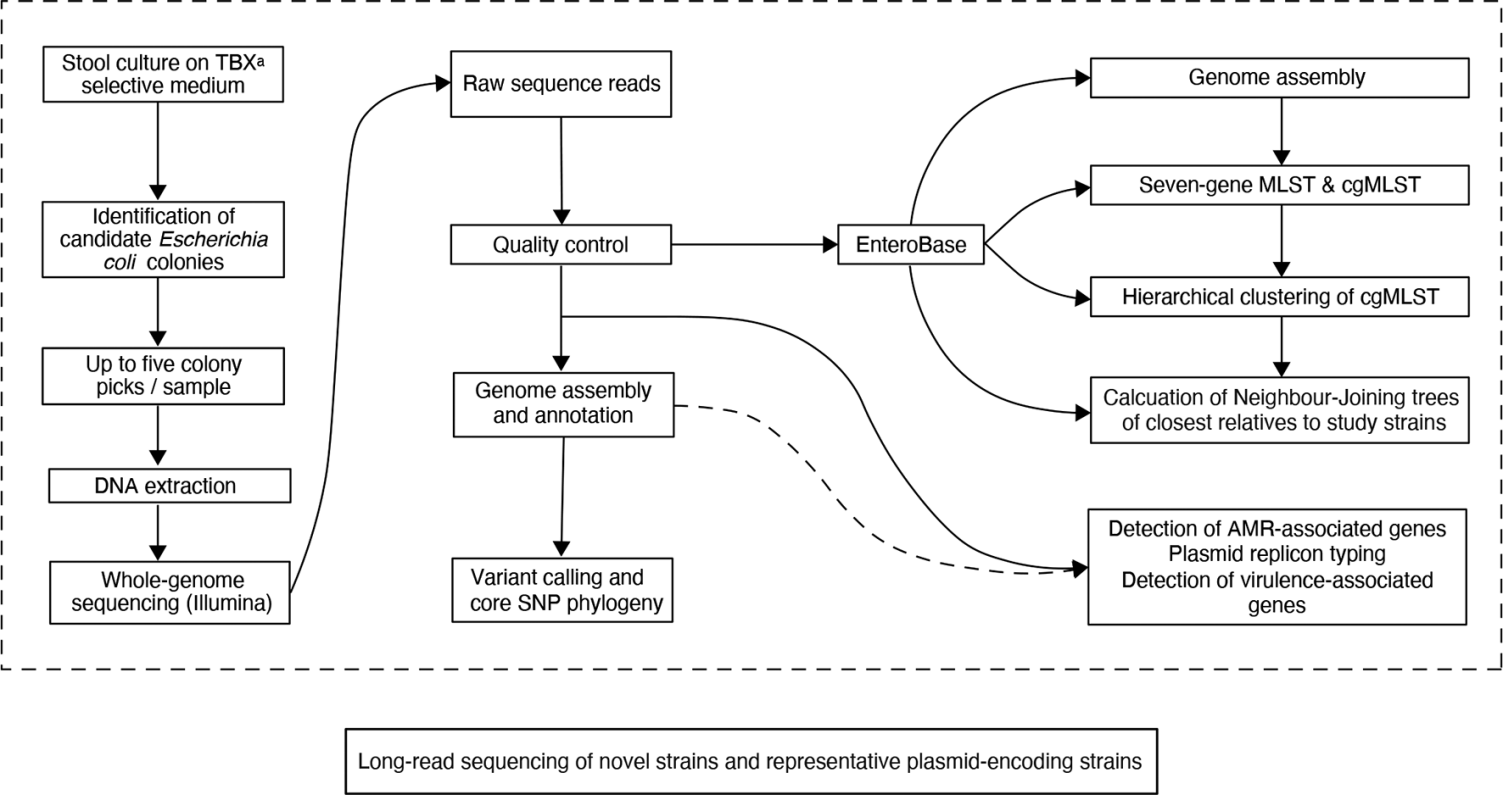
Study sample-processing flow diagram.

Troops of monkeys were observed and followed. We collected single freshly passed formed stool specimens from 43 visibly healthy individuals (38 adults, 5 juveniles; 24 females, 11 males, 8 of undetermined sex), drawn from four species: *Erythrocebus patas* (patas monkey), *Papio papio* (Guinea baboon), *Chlorocebus sabaeus* (green monkey) and *Piliocolobus badius* (Western colobus monkey). Stool samples were immediately placed into sterile falcon tubes, taking care to collect portions of stool material that had not touched the ground, then placed on dry ice and stored at 80 °C within 6 h (Fig. 2).

### Microbiological processing

For the growth and isolation of *E. coli *, 0.1–0.2 g aliquots were taken from each stool sample into 1.5 ml microcentrifuge tubes under aseptic conditions. To each tube, 1 ml of physiological saline (0.85 %) was added and the saline-stool samples were vortexed for 2 min at 4, 200 r.p.m. The homogenized samples were taken through four tenfold serial dilutions and a 100 µl aliquot from each dilution was spread on a plate of tryptone-bile-X-glucoronide agar using the cross-hatching method. Plates were incubated at 37 °C for 18–24 h in air. Colony counts were performed for each serial dilution, counting translucent colonies with blue-green pigmentation and entire margins as *E. coli *. Up to five colonies from each sample were sub-cultured on MacConkey agar at 37 °C for 18–24 h and then stored in 20 % glycerol broth at −80 °C. Previous studies have shown that sampling five colonies provides a 99.3 % chance of recovering at least one of the dominant genotypes present in a single stool specimen [23, 24].

### Genomic DNA extraction

A single colony from each subculture was picked into 1 ml Luria–Bertani broth and incubated overnight at 37 °C. Broth cultures were spun at 3, 500 r.p.m. for 2 min and lysed using lysozyme, proteinase K, 10 % SDS and RNase A in Tris EDTA buffer (pH 8.0). Suspensions were placed on a thermomixer with vigorous shaking at 1600 r.p.m., first at 37 °C for 25 min and subsequently at 65 °C for 15 min. DNA was extracted using solid-phase reversible immobilisation magnetic beads (Becter Coulter, Brea, CA, USA), precipitated with ethanol, eluted in Tris-Cl and evaluated for protein and RNA contamination using A_260_/A_280_ and A_260_/A_230_ ratios on the NanoDrop 2000 Spectrophotometer (Fisher Scientific, Loughborough, UK). DNA concentrations were measured using the Qubit HS DNA assay (Invitrogen, MA, USA). DNA was stored at −20 °C.

### Illumina sequencing

Whole-genome sequencing was carried out on the Illumina NextSeq 500 platform (Illumina, San Diego, CA, USA). We used a modified Nextera XT DNA protocol for the library preparation (File S1). The genomic DNA was normalized to 0.5 ng µl^−1^ with 10 mM Tris-HCl prior to the library preparation. The pooled library was run at a final concentration of 1.8 pM on a mid-output flow cell (NSQ 500 Mid Output KT v2 300 cycles; Illumina Catalogue No. FC-404–2003) following the Illumina recommended denaturation and loading parameters, which included a 1 % PhiX spike (PhiX Control v3; Illumina Catalogue FC-110–3001). The data was uploaded to BaseSpace (http://www.basespace.illumina.com) and then converted to FASTQ files.

### Oxford nanopore sequencing

We used the rapid barcoding kit (Oxford Nanopore Catalogue No. SQK-RBK004) to prepare libraries according to the manufacturer’s instructions. We used 400 ng DNA for library preparation and loaded 75 µl of the prepared library on an R9.4 MinION flow cell. The size of the DNA fragments was assessed using the Agilent 2200 TapeStation (Agilent Catalogue No. 5067–5579) before sequencing. The concentration of the final library pool was measured using the Qubit high-sensitivity DNA assay (Invitrogen, MA, USA).

### Genome assembly and phylogenetic analysis

Sequences were analysed on the Cloud Infrastructure for Microbial Bioinformatics [25]. Paired-end short-read sequences were concatenated, then quality-checked using FastQC v0.11.7 [26]. Reads were assembled using Shovill (https://github.com/tseemann/shovill) and assemblies assessed using QUAST v 5.0.0, de6973bb [27]. Draft bacterial genomes were annotated using Prokka v 1.13 [28]. Multi-locus sequence types were called from assemblies according to the Achtman scheme using the mlst software (https://github.com/tseemann/mlst) to scan alleles in PubMLST (https://pubmlst.org/) [29]. Novel STs were assigned by EnteroBase – an online integrated software environment, which routinely retrieves short-read *E. coli* sequences from the public domain, or using user-uploaded short reads, *de novo* assembles these and assigns seven-allele MLST (ST) and phylogroups from genome assemblies using standardized pipelines [30]. EnteroBase assigns new allele IDs or STs in the event of a locus being discovered with a novel allele. Snippy v4.3.2 (https://github.com/tseemann/snippy) was used for variant calling and core genome alignment, including reference genome sequences representing the major phylogroups of *E. coli* and *Escherichia fergusonii* as an outgroup (File S2b). We used Gubbins (Genealogies Unbiased By recomBinations In Nucleotide Sequences) to detect and remove recombinant regions of the core genome alignment [31]. RAxML v 8.2.4 [32] was used for maximum-likelihood phylogenetic inference from this masked alignment based on a general time-reversible nucleotide substitution model with 1, 000 bootstrap replicates. The phylogenetic tree was visualized using Mega v. 7.2 [33] and annotated using Adobe Illustrator v 23.0.3 (Adobe, San Jose, CA, USA). Pair-wise SNP distances between genomes were computed from the core-gene alignment using snp-dists v0.6 (https://github.com/tseemann/snp-dists).

### Population structure and analysis of gene content

Merged short reads were uploaded to EnteroBase [30], where we used the Hierarchical Clustering (HierCC) algorithm to assign our genomes from non-human primates to HC1100 clusters, which in *E. coli* correspond roughly to the clonal complexes seen in seven-allele MLST. Core genome MLST (cgMLST) profiles based on the typing of 2, 512 core loci for *E. coli* facilitates single-linkage hierarchical clustering according to fixed core genome MLST (cgMLST) allelic distances, based on cgMLST allelic differences. Thus, cgST HierCC provides a robust approach to analyse population structures at multiple levels of resolution. The identification of closely related genomes using HierCC has been shown to be 89 % consistent between cgMLST and SNPs [34]. Neighbour-joining trees were reconstructed with NINJA – a hierarchical clustering algorithm for inferring phylogenies that is capable of scaling to inputs larger than 100, 000 sequences [35].

ARIBA v2.12.1 [36] was used to search short reads against the Virulence Factors Database [37] (VFDB-core) (virulence-associated genes), ResFinder (AMR) [38] and PlasmidFinder (plasmid-associated genes) [39] databases (both ResFinder and PlasmidFinder databases downloaded 29 October 2018). Percentage identity of ≥90 % and coverage of ≥70 % of the respective gene length were taken as a positive result. Analyses were performed on assemblies using ABRicate v 0.8.7 (https://github.com/tseemann/abricate). A heat map of detected virulence- and AMR-associated genes was plotted on the phylogenetic tree using ggtree and phangorn in RStudio v 3.5.1.

We searched EnteroBase for all *E. coli* strains isolated from humans in the Gambia (*n*=128), downloaded the genomes and screened them for resistance genes using ABRicate v 0.9.8. Assembled genomes for isolates that clustered with our colibactin-encoding ST73, ST127 and ST681 isolates were downloaded and screened for the colibactin operon using ABRicate’s VFDB database (accessed 28 July 2019). Assemblies reported to contain colibactin genes were aligned against the colibactin-encoding *E. coli* IHE3034 reference genome (NCBI accession: GCA_000025745.1) using minimap2 2.13-r850. BAM files were visualized in Artemis Release 17.0.1 [40] to confirm the presence of the *pks* genomic island, which encodes the colibactin operon (*clbA-S*).

### Hybrid assembly and analysis of plasmids and phages

Base-called FASTQ files were concatenated into a single file and demultiplexed into individual FASTQ files based on barcodes, using the qcat python command-line tool v 1.1.0 (https://github.com/nanoporetech/qcat). Hybrid assemblies of the Illumina and nanopore reads were created with Unicycler [41]. The quality and completion of the hybrid assemblies were assessed with QUAST v 5.0.0, de6973bb and CheckM [27, 42]. Hybrid assemblies were interrogated using ABRicate PlasmidFinder and annotated using Prokka [28]. Plasmid sequences were visualized in Artemis using coordinates from ABRicate. Prophage identification was carried out using the phage search tool, PHASTER [43].

### Antimicrobial susceptibility

We determined the MICs of amikacin, trimethoprim, sulfamethoxazole, ciprofloxacin, cefotaxime and tetracycline for the isolates from non-human primates using agar dilution [44]. Twofold serial dilutions of each antibiotic were performed in molten Mueller–Hinton agar (Oxoid, Basingstoke, UK), from 32 mg l^−1^ to 0.03 mg l^−1^ (512 mg l^−1^ to 0.03 mg l^−1^ for sulfamethoxazole), using *E. coli* NCTC 10418 as control. MICs were performed in duplicate and interpreted using breakpoint tables from the European Committee on Antimicrobial Susceptibility Testing v. 9.0, 2019 (http://www.eucast.org).

### Statistical analysis

We prepared a table to show the phylotype distribution per individual and visualized this as a heatmap in RStudio v 3.5.1. We carried out Fisher’s exact tests to assess possible associations between the sampling site or non-human primate species and the phylogroups of *E. coli* that were observed using STATA version 14.2. We based our calculations on the assumption of independence across the observed phylogroups, i.e. the finding of one phylogroup does not predict or preclude the occurrence of another. Prior to the association tests, replicate phylogroups arising from copies of the same ST from a single individual were dropped from the analysis.

We calculated co-occurrence of the detected resistance genes among the study isolates and visualised this as a heatmap in RStudio v 3.5.1. In addition, we generated contingency tables to display the correlation between the phenotypic results and the detected resistance genes among the study isolates and calculated the percentage concordance between the genotypic and phenotypic resistances.

## Results

### Study population

Twenty-four of 43 samples (56 %) showed growth indicative of *E. coli *, yielding a total of 106 colonies. The isolates were designated by the primate species and the site from which they were sampled as follows: *Chlorocebus sabaeus*, ‘Chlos’; *Papio papio*, ‘Pap’; *Piliocolobus badius*, ‘Prob’; Abuko Nature Reserve, ‘AN’; Bijilo Forest Park, ‘BP’; Kartong village, ‘K’; Kiang West National Park, ‘KW’; Makasutu Cultural Forest, ‘M’; and River Gambia National Park, ‘RG’. After genome sequencing, five isolates [PapRG-04, (*n*=1); PapRG-03 (*n*=1); ChlosRG-12 (*n*=1); ChlosAN-13 (*n*=1); ProbAN-19 (*n*=1)] were excluded due to low depth of coverage (<20×), leaving 101 genomes for subsequent analysis (Table 1 and File S2a).

**Table 1.** Study isolates

Name	Source	Individual sampling no.	Colony-pick	Sampling site	ST
PapRG-03–1	*Papio papio*	3	1	River Gambia national park	336
PapRG-03–2	*Papio papio*	3	2	River Gambia national park	336
PapRG-03–3	*Papio papio*	3	3	River Gambia national park	336
PapRG-03–4	*Papio papio*	3	4	River Gambia national park	336
PapRG-03–5	*Papio papio*	3	5	River Gambia national park	336
PapRG-04–1	*Papio papio*	4	1	River Gambia national park	1665
PapRG-04–2	*Papio papio*	4	2	River Gambia national park	1204
PapRG-04–4	*Papio papio*	4	3	Makasutu cultural forest	8826
PapRG-04–5	*Papio papio*	4	4	Makasutu cultural forest	1204
PapRG-05–2	*Papio papio*	5	1	Makasutu cultural forest	1431
PapRG-05–3	*Papio papio*	5	2	Makasutu cultural forest	99
PapRG-05–4	*Papio papio*	5	3	Makasutu cultural forest	6316
PapRG-05–5	*Papio papio*	5	4	Makasutu cultural forest	1431
PapRG-06–1	*Papio papio*	6	1	Makasutu cultural forest	4080
PapRG-06–2	*Papio papio*	6	2	Makasutu cultural forest	2521
PapRG-06–3	*Papio papio*	6	3	Makasutu cultural forest	8827
PapRG-06–4	*Papio papio*	6	4	Makasutu cultural forest	1204
PapRG-06–5	*Papio papio*	6	5	River Gambia national park	8525
ProbRG-07–1	*Piliocolobus badius*	7	1	River Gambia national park	73
ProbRG-07–2	*Piliocolobus badius*	7	2	River Gambia national park	73
ProbRG-07–3	*Piliocolobus badius*	7	3	River Gambia national park	73
ProbRG-07–4	*Piliocolobus badius*	7	4	River Gambia national park	73
ProbRG-07–5	*Piliocolobus badius*	7	5	River Gambia national park	73
ChlosRG-12–1	*Chlorocebus sabaeus*	12	1	River Gambia national park	8824
ChlosRG-12–2	*Chlorocebus sabaeus*	12	2	River Gambia national park	196
ChlosRG-12–3	*Chlorocebus sabaeus*	12	3	River Gambia national park	196
ChlosRG-12–5	*Chlorocebus sabaeus*	12	4	River Gambia national park	40
ChlosAN-13–1	*Chlorocebus sabaeus*	13	1	Abuko Nature Reserve	8526
ChlosAN-13–2	*Chlorocebus sabaeus*	13	2	Abuko Nature Reserve	8550
ChlosAN-13–4	*Chlorocebus sabaeus*	13	3	Abuko Nature Reserve	1973
ChlosAN-13–5	*Chlorocebus sabaeus*	13	4	Abuko Nature Reserve	1973
PapAN-14–1	*Papio papio*	14	1	Abuko Nature Reserve	2076
PapAN-14–2	*Papio papio*	14	2	Abuko Nature Reserve	939
PapAN-14–3	*Papio papio*	14	3	Abuko Nature Reserve	226
PapAN-14–4	*Papio papio*	14	4	Abuko Nature Reserve	226
PapAN-14–5	*Papio papio*	14	5	Abuko Nature Reserve	226
PapAN-15–1	*Papio papio*	15	1	Abuko Nature Reserve	226
PapAN-15–2	*Papio papio*	15	2	Abuko Nature Reserve	5073
PapAN-15–3	*Papio papio*	15	3	Abuko Nature Reserve	226
PapAN-15–4	*Papio papio*	15	4	Abuko Nature Reserve	126
PapAN-15–5	*Papio papio*	15	5	Abuko Nature Reserve	8823
ChlosAN-17–1	*Chlorocebus sabaeus*	17	1	Abuko Nature Reserve	681
ChlosAN-17–2	*Chlorocebus sabaeus*	17	2	Abuko Nature Reserve	362
ChlosAN-17–3	*Chlorocebus sabaeus*	17	3	Abuko Nature Reserve	681
ChlosAN-17–4	*Chlorocebus sabaeus*	17	4	Abuko Nature Reserve	681
ChlosAN-18–1	*Chlorocebus sabaeus*	18	1	Abuko Nature Reserve	681
ChlosAN-18–2	*Chlorocebus sabaeus*	18	2	Abuko Nature Reserve	681
ChlosAN-18–3	*Chlorocebus sabaeus*	18	3	Abuko Nature Reserve	681
ChlosAN-18–4	*Chlorocebus sabaeus*	18	4	Abuko Nature Reserve	681
ChlosAN-18–5	*Chlorocebus sabaeus*	18	5	Abuko Nature Reserve	349
ProbAN-19–2	*Piliocolobus badius*	19	1	Abuko Nature Reserve	8825
ChlosBP-21–1	*Chlorocebus sabaeus*	21	1	Bijilo forest park	677
ChlosBP-21–2	*Chlorocebus sabaeus*	21	2	Bijilo forest park	677
ChlosBP-21–3	*Chlorocebus sabaeus*	21	3	Bijilo forest park	677
ChlosBP-21–4	*Chlorocebus sabaeus*	21	4	Bijilo forest park	677
ChlosBP-21–5	*Chlorocebus sabaeus*	21	5	Bijilo forest park	677
ChlosBP-23–1	*Chlorocebus sabaeus*	23	2	Bijilo forest park	8527
ChlosBP-23–2	*Chlorocebus sabaeus*	23	3	Bijilo forest park	8527
ChlosBP-23–3	*Chlorocebus sabaeus*	23	4	Bijilo forest park	3306
ChlosBP-24–1	*Chlorocebus sabaeus*	24	1	Bijilo forest park	73
ChlosBP-24–2	*Chlorocebus sabaeus*	24	2	Bijilo forest park	73
ChlosBP-24–3	*Chlorocebus sabaeus*	24	3	Bijilo forest park	73
ChlosBP-24–4	*Chlorocebus sabaeus*	24	4	Bijilo forest park	73
ChlosBP-24–5	*Chlorocebus sabaeus*	24	5	Bijilo forest park	73
ChlosBP-25–1	*Chlorocebus sabaeus*	25	1	Bijilo forest park	73
ChlosBP-25–2	*Chlorocebus sabaeus*	25	2	Bijilo forest park	73
ChlosBP-25–3	*Chlorocebus sabaeus*	25	3	Bijilo forest park	73
ChlosBP-25–4	*Chlorocebus sabaeus*	25	4	Bijilo forest park	73
ChlosBP-25–5	*Chlorocebus sabaeus*	25	5	Bijilo forest park	73
ChlosM-29–1	*Chlorocebus sabaeus*	29	1	Makasutu cultural forest	1873
ChlosM-29–2	*Chlorocebus sabaeus*	29	2	Makasutu cultural forest	1873
PapM-31–1	*Papio papio*	31	1	Makasutu cultural forest	2800
PapM-31–2	*Papio papio*	31	2	Makasutu cultural forest	135
PapM-31–3	*Papio papio*	31	3	Makasutu cultural forest	5780
PapM-31–4	*Papio papio*	31	4	Makasutu cultural forest	1727
PapM-31–5	*Papio papio*	31	5	Makasutu cultural forest	5780
PapM-32–1	*Papio papio*	32	2	Makasutu cultural forest	8532
PapM-32–2	*Papio papio*	32	3	Makasutu cultural forest	212
PapM-32–3	*Papio papio*	32	4	Makasutu cultural forest	212
PapM-32–4	*Papio papio*	32	5	Makasutu cultural forest	212
PapM-32–5	*Papio papio*	32	6	Makasutu cultural forest	212
PapM-33–1	*Papio papio*	33	1	Makasutu cultural forest	8533
PapM-33–2	*Papio papio*	33	2	Makasutu cultural forest	8533
PapM-33–3	*Papio papio*	33	3	Makasutu cultural forest	8533
PapM-33–4	*Papio papio*	33	4	Makasutu cultural forest	38
PapM-33–5	*Papio papio*	33	5	Makasutu cultural forest	8533
PapM-34–1	*Papio papio*	34	1	Makasutu cultural forest	676
PapM-34–2	*Papio papio*	34	2	Makasutu cultural forest	676
PapM-34–3	*Papio papio*	34	3	Makasutu cultural forest	676
PapM-34–4	*Papio papio*	34	4	Makasutu cultural forest	676
PapM-36–1	*Papio papio*	36	1	Makasutu cultural forest	8535
PapM-36–2	*Papio papio*	36	2	Makasutu cultural forest	8535
PapKW-44–1	*Papio papio*	44	1	Kiang West national park	442
PapKW-44–2	*Papio papio*	44	2	Kiang West national park	442
PapKW-44–3	*Papio papio*	44	3	Kiang West national park	442
PapKW-44–4	*Papio papio*	44	4	Kiang West national park	442
ProbK-45–1	*Piliocolobus badius*	45	1	Kartong village	127
ProbK-45–2	*Piliocolobus badius*	45	2	Kartong village	127
ProbK-45–3	*Piliocolobus badius*	45	3	Kartong village	127
ProbK-45–4	*Piliocolobus badius*	45	4	Kartong village	127
ProbK-45–5	*Piliocolobus badius*	45	5	Kartong village	127

### Distribution of sequence types and phylogroups

**Fig. 3.**
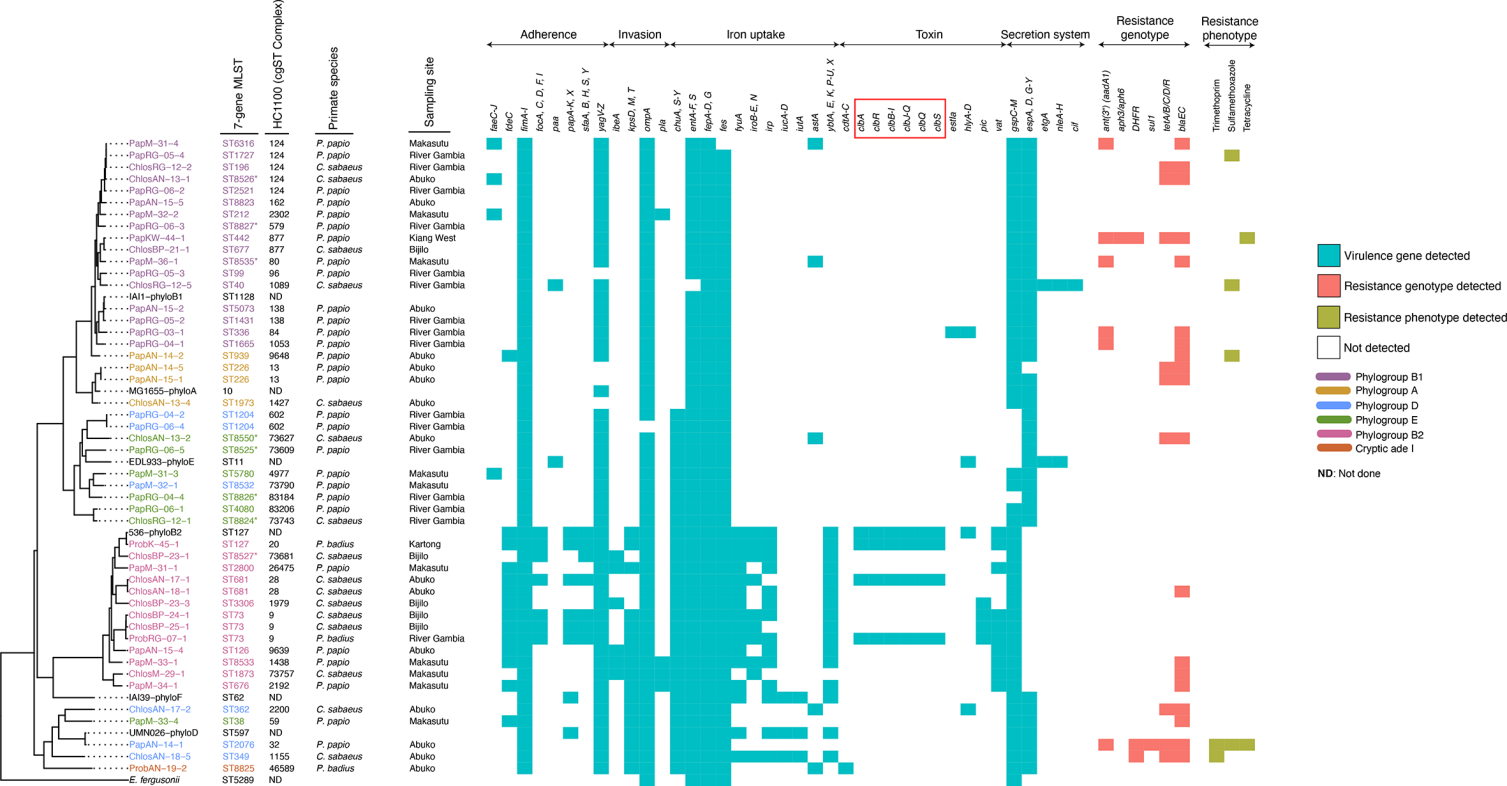
Plot showing the maximum-likelihood phylogeny of the study isolates overlaid with the prevalence of putative virulence genes and resistance-associated genes, as well as the phenotypic antimicrobial resistance among the study isolates. The tree was reconstructed based on non-repetitive core SNPs calculated against the *E. coli* K-12 reference strain (NCBI accession: NC_000913.3), using RAxML with 1000 bootstrap replicates. *E. coli* MG1655 was used as the reference and *E. fergusonii* as the outroot species. Recombinant regions were removed using Gubbins [31]. The tip labels indicate the sample IDs, with the respective *in silico* Achtman STs and HC1100 (cgST complexes) indicated next to the tip labels. Both the sample IDs and the STs (Achtman) are colour-coded to indicate the various phylogroups as indicated. Novel STs (Achtman) are indicated by an asterisk (*). *Escherichia fergusonii* and the *E. coli* reference genomes representing the major *E. coli* phylogroups are in black. Primate species are indicated by strain names as follows: *Chlorocebus sabaeus*, ‘Chlos’; *Papio papio*, ‘Pap’; *Piliocolobus badius*, ‘Prob’. These strain designations are also used in annotating the plot next to the tree. The sampling sites are indicated as follows: BP, Bijilo forest park; KW, Kiang-West National park; RG, River Gambia National Park; M, Makasutu Cultural forest; AN, Abuko Nature reserve; K, Kartong village. These site designations are also used in annotating the plot next to the tree. Cocolonising seven-allele (Achtman) STs in single individuals are shown by the prefix of the strain names depicting the colony as 1, 2 up to 5. We do not show multiple colonies of the same Achtman ST recovered from a single individual. In such cases, only one representative is shown. Virulence genes are grouped according to their function, with genes encoding the colibactin genotoxin highlighted with a red box. The full names of virulence factors are provided in File S7. The resistance-associated genes detected among the study isolates and the class of antibiotic to which they encode resistance are as follows: *ant*(3′′) (*aadA1*) and *aph3/aph6*, aminoglycosides; DHFR, trimethoprim; *sul1*, sulphonamides; *tetA/B/C/D/R*, tetracyclines; *bla*EC, beta-lactamase (penicillinase-type).

**Fig. 4.**
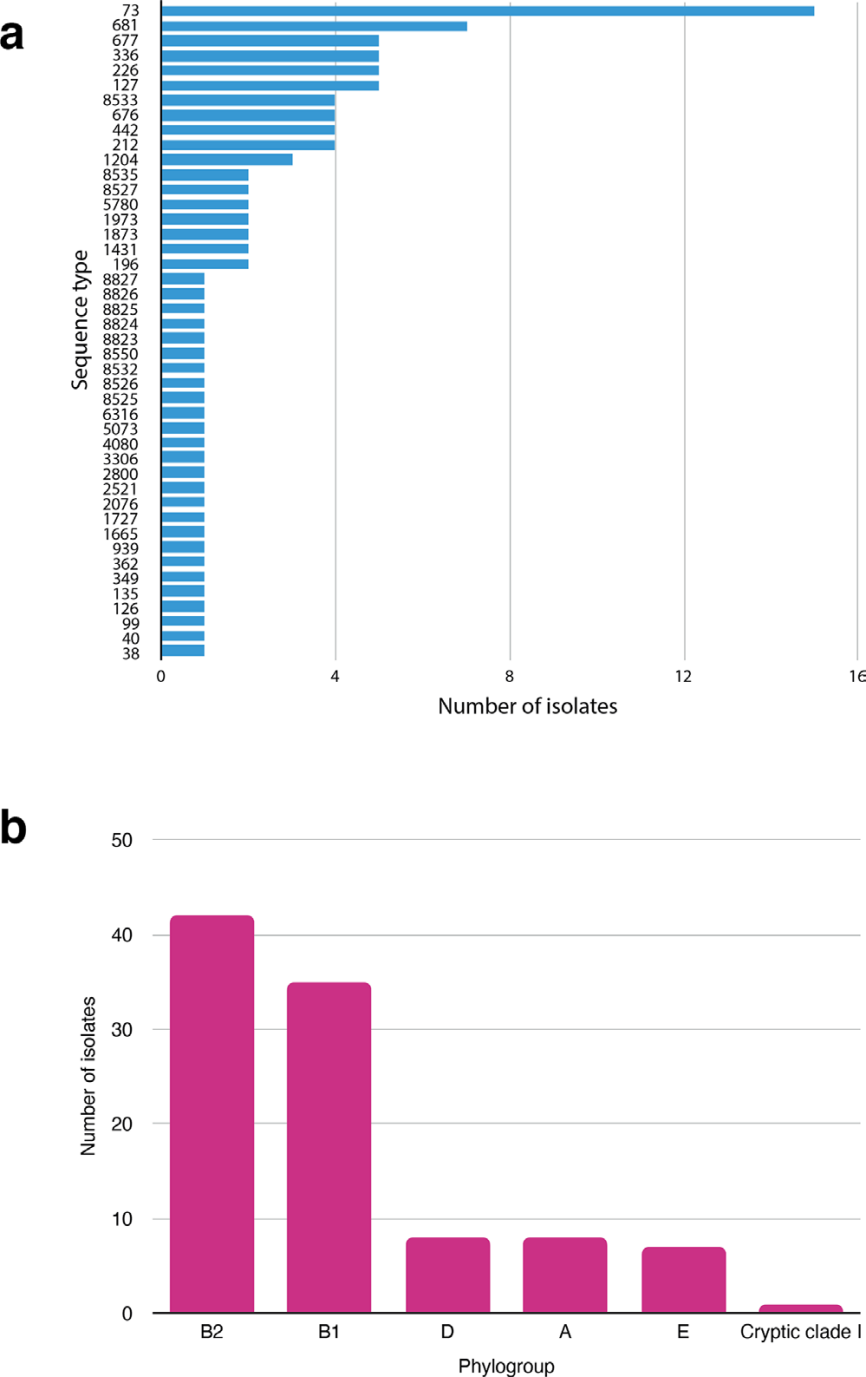
(a) Distribution of sequence types (STs) among the study isolates. (b) Distribution of phylotypes among the study isolates.

We recovered 43 seven-allele sequence types (ten of them novel), spanning five of the eight known phylogroups of *E. coli* and comprising 38 core-genome MLST complexes (Figs. 3 and 4). The majority of strains belonged to phylogroup B2 (42/101, 42 %) – which encompasses strains that cause extraintestinal infections in humans (ExPEC strains) [4, 45, 46] – followed by B1 (35/101, 35 %), A and D (8/101, 8 % each), E (7/101, 7 %) and cryptic clade I (1/101, 1 %). Among the study isolates, we found several STs associated with extraintestinal infections and/or AMR in humans: ST73, ST681, ST127, ST226, ST336, ST349 [47–49]. We did not find any significant associations between the primate species and the distribution of phylogroups (*P*=0.17), nor between the sampling sites and phylogroups (*P*=0.44). The distribution of phylotype per individual is presented in Fig. S4.

### Prevalence of virulence factors

We detected a total of 146 virulence factors among the study isolates (Fig. 3 and File S7). The following virulence factors were largely conserved across most of the study isolates: the enterobactin-associated cluster of genes (*fepA-D, G* and *entA-F, S*), type I fimbriae (*fimA-I*) and the fimbria-associated genes (*yagV-Z*). However, iron-acquisition genes (*chuA, S-Y*) appeared to be more prevalent in strains belonging to phylogroups B2, D and E. In general, we detected a higher prevalence of virulence genes in strains belonging to phylogroup B2, compared to those from phylogroups A, B1, D and E (Fig. 3). These included additional siderophore-encoding genes (*ybt, fyu* and *irp*), capsular antigens (*kpsM1/D*), salmochelin (*iroN/C/B/D/E*), P, S and F1C fimbriae genes (*papC, D, I-K,* X, *focI/C/F* and *sfaY/B,* respectively) and the adherence factor protein gene (*fde*C) – representing colonization and fitness factors associated with extraintestinal disease in humans.

A subset of the B2 strains (13/42, 31 %), belonging to STs 73, 681 and 127, carried the *pks* genomic island (*clbA-S*), which encodes the DNA alkylating genotoxin, colibactin (Fig. 3, red box). Colibactin-encoding *E. coli* frequently cause colorectal cancer, urosepsis, bacteraemia and prostatitis and commonly carry other virulence factors such as siderophores and toxins [50–52]. Also, all the ST73 (phylogroup B2) strains carried genes encoding the Serin protease autotransporter (*pic*) and 79 %(33/42) of the B2 strains possessed the vacuolating autotransporter (*vat*) toxins.

Besides the B2 strains, we also detected toxins associated with intestinal and extraintestinal disease in humans among strains from other phylogroups (File S7): in particular the heat-stable enterotoxin 1 (*astA*) occurred in five isolates overall (two phylogroup B1 and one each of phylogroups E, D and the *Escherichia* cladeI); the haemolyin genes (*hlyB-D*) were detected in a single Guinea baboon (PapRG-03, phylogroup B1); the invasion of brain endothelium gene (*ibe*A) – responsible for neonatal meningitis in humans – was observed in six Guinea baboon isolates (all belonging to phylogroup B2) derived from PapM33 and PapM-34 and one Green monkey (ChlosM-29).

### Within-host genomic diversity

Thirteen individuals were colonized by two or more STs and nine by two or more phylogroups (File S2a). Five colony picks from a single Guinea baboon (PapRG-06) yielded five distinct STs, two of which are novel. Two green monkeys sampled from Bijilo (ChlosBP-24 and ChlosBP-25) shared an identical ST73 genotype (zero SNP difference between the two genomes), while two Guinea baboons from Abuko shared an ST226 strain (zero SNP difference) – documenting transmission between monkeys of the same species.

In seventeen monkeys, we observed a cloud of closely related genotypes (separated by 1–5 SNPs, Table 2a) from each strain, suggesting evolution within the host after acqusition of the strain. However, in two individuals, pair-wise SNP distances between genotypes from the same ST were susbtantial enough (25 SNPs and 77 SNPs) to suggest multiple acquisitions of each strain (Table 2b). Reeves *et al*. [53] estimated a mutation rate of 1.1 SNP per genome per year from characterizing fourteen ST73 strains isolated from a single family over three years. Based on this data, with the assumption that equal rates of mutation occurred in both genomes, we can infer about 11–35 years of divergence for these strains. Thus, it is implausible that these strains represent within-host diversity and persistence in the two hosts, judging by the lifespan of a green monkey in the wild (averaged at 17 years).

**Table 2.** (a) Within-host SNP diversity between multiple genomes of the same ST recovered from the same monkey. (b) Within-host diversity in green monkey 25 (ChlosBP-25)

(a) Sample ID	STs (colonies per ST)	Pair-wise SNP distances between multiple colonies of the same ST	Comment(s)
PapRG-03	336 (*n*=5)	0–2	
PapRG-04	1204 (*n*=2)	4	
PapRG-05	1431 (*n*=2)	0	
ProbRG-07	73 (*n*=5)	0–1	
ChlosRG-12	196 (*n*=2)	25	
PapAN-14	226 (*n*=3)	1	
PapAN-15	226 (*n*=2)	1	
ChlosAN-17	681 (*n*=3)	0–3	
ChlosAN-18	681 (*n*=4)	0	
ChlosBP-21	677 (*n*=4)	5	
ChlosBP-23	8527 (*n*=2)	0	
ChlosBP-24	73 (*n*=5)	0–5	
ChlosBP-25	73 (*n*=5)	0–79	Please see Table 2b
PapM-32	212 (*n*=4)	0	
PapM-33	8533 (*n*=4)	0–4	
PapM-34	676 (*n*=4)	0–1	
PapM-36	8535 (*n*=2)	0–1	
PapKW-44	442 (*n*=4)	1–2	
ProbK-45	127 (*n*=5)	0–4	

(b) Sample ID	Clone designation		
**ChlosBP-25**			
ChlosBP-25-1	1		
ChlosBP-25-2	2		
ChlosBP-25-3	2		
ChlosBP-25-4	2		
ChlosBP-25-5	3		
**Pair-wise SNP distances between clones**			
	Clone 1	Clone 2	Clone 3
Clone 1	0	12	79
Clone 2	12	0	67
Clone 3	79	67	0

### Population structure of simian *E. coli* isolates

We identified the closest neighbours of all strains from our study (Table 3). Our results suggest, in some cases, recent interactions between humans or livestock and non-human primates. However, we also found a diversity of strains specific to the non-human primate niche. Hierarchical clustering analysis revealed that simian isolates from ST442 and ST349 (Achtman) – sequence types that are associated with virulence and AMR in humans [49, 54] – were closely related to human clinical isolates, with differences of 50 alleles and seven alleles in the core-genome MLST scheme, respectively (Figs S1 and S2). Similarly, we found evidence of recent interaction between simian ST939 isolates and strains from livestock (Fig. S3) – with 40 cgMLST alleles (<40 SNPs) separating the two genomes, representing less than 18 years of divergence. Conversely, simian ST73, ST127 and ST681 isolates were genetically distinct from human isolates from these sequence types (Figs S5–S7). The only multi-drug resistant isolate (PapAN-14–1) from ST2076 was, however, closely related to an environmental isolate recovered from water (Fig. S8).

**Table 3.** Genomic relationship between study isolates and publicly available *E. coli* genomes

Seven-allele ST	HC100 subgroups	Non-human primate host	Closest neighbours' source	Neighbours' country of isolation	Allelic distance
**349**	–	*Chlorocebus sabaeus* 18	Human (bloodstream infection)	Canada	7
**2076**	–	*Papio papio* 14	Environment (water)	Unknown	25
**939**	–	*Papio papio* 14	Livestock	US	40
**442**	–	*Papio papio* 44	Human	China	50
**2800**	–	*Papio papio* 31	Unknown	Vietnam	59
**1973**	–	*Chlorocebus sabaeus* 13	Unknown	Unknown	64
**8533**	–	*Papio papio* 33	Environment (water)	Unknown	69
**6316**	–	*Papio papio* 05	Human	Kenya	97
**1727**	–	*Papio papio* 34	Human	Kenya	98
**676**	–	*Papio papio* 34	Human (bloodstream infection)	UK	98
**8823**	–	*Papio papio* 15	Rodent (guinea pig)	Kenya	101
**1431**	–	*Papio papio* 05	Human	US	109
**5073**	–	*Papio papio* 15	Human	US	112
**226**	73 641	*Papio papio* 14	Human	Tanzania	112
**8827**	–	*Papio papio* 06	Human	Unknown	122
**1204**	83 197	*Papio papio* 04	Livestock	Japan	127
**1204**	83 197	*Papio papio* 04	Livestock	Japan	130
**677**	–	*Chlorocebus sabaeus* 21	Human	US	132
**40**	–	*Chlorocebus sabaeus* 12	Human	UK	137
**1204**	83 164	*Papio papio* 06	Livestock	Japan	173
**99**	–	*Papio papio* 05	Human	UK	180
**362**	–	*Chlorocebus sabaeus* 17	Food	Kenya	180
**8825**	–	*Piliocolobus badius* 19	Human	France	189
**336**	–	*Papio papio* 03	Poultry	Kenya	189
**73**	–	*Chlorocebus sabaeus* 24	Human	Sweden	189
**196**	–	*Chlorocebus sabaeus* 12	Human	Sweden	197
**2521**	–	*Papio papio* 06	Livestock	US	201
**127**		*Pioliocolobus badius* 45	Companion animal	US	229
**681**		*ChlosAN* 17	Human	Norway	251
**38**	–	*Papio papio* 33	human	UK	265
**135**	–	*Papio papio* 31	Poultry	US	281
**8824**	–	*Chlorocebus sabaeus* 12	Environmental*	US	296
**226**	100 039	*Papio papio* 14	Human	Sri Lanka	318
**8527**	–	*Chlorocebus sabaeus* 23	Human	Kenya	323
**8535**	–	*Papio papio* 36	Environmental (soil)	US	368
**1665**	–	*Papio papio* 04	Livestock	UK	371
**4080**	–	*Papio papio* 06	Human	Denmark	507
**8526**	–	*Chlorocebus sabaeus* 13	Livestock	US	708
**8532**	–	*Papio papio* 32	Non-human primate	Gambia (PapM-31–3)	1102
**8826**	–	*Papio papio* 04	Livestock	Mozambique	1255
**8525**	–	*Papio papio* 06	Livestock/companion animal	Switzerland	1659
**1873**	–	*Chorocebus sabaeus* 29	Environment	US	1685
**8550**	–	*Chlorocebus sabaeus* 13	Unknown	Unknown	2006

*Source details unknown.

Isolates from humans were recovered from stools, except where indicated otherwise.

Five isolates were >1, 000 alleles away in the core-genome MLST scheme from anything in EnteroBase (Figs S9 and S10). Four of these were assigned to novel sequence types in the seven-allele scheme (Achtman) (ST8550, ST8525, ST8532, ST8826), while one belonged to ST1873, which has only two other representatives in EnteroBase: one from a species of wild bird from Australia (*Sericornis frontalis*); the other from water. Besides, ST8550, ST8525, ST8532 and ST8826 belonged to novel HierCC 1100 groups (cgST complexes), indicating that they were distinct from any other publicly available *E. coli* genomes.

Besides our study isolates, there were 94 *E. coli* genomes sourced from non-human primates from the rest of the world within EnteroBase: the USA (83), Uganda (6), Kenya (4), Mexico (1). A total of 52 STs were found among these primates from other parts of the world (Fig. S11a), four of which were also found among our study isolates (ST 73, ST127, ST681 and ST939). As observed in our monkey isolates, the most common ST among primates from the rest of the world was ST73 (11 %). Also, most of the non-Gambian primate isolates belonged to phylogroup B2 (41 %) and B1 (21 %), consistent with what we found in our study population (Fig. S11b). Hierarchical clustering based on cgMLST types revealed clustering patterns that were largely consistent with the phylotype designations to which the primate isolates belonged. No discernible segregation of primate *E. coli* phylotypes based on geography was observed.

### Prevalence of AMR-associated genes

We observed a modest prevalence of genotypic antimicrobial resistance in our study population. The AMR-associated genotypes we found among the monkey isolates included *bla*EC (beta-lactamase, penicillinase-type), *ant(3′) (aadA1*) (streptomycin and spectinomycin), *aph3/aph6* (neomycin and kanamycin), DHFR (trimethoprim), *sul1* (sulphonamides) and *tetA/B/C/D/R* (tetracyclines) (Fig. 3). A total of 22 isolates encoded resistance genes to a single antibiotic agent; 22 to two antibiotic classes and three isolates to three or more antibiotic classes. Pair-wise co-occurrence of AMR-associated genes in the same genome was sparse. The most common gene network was *bla*EC*-tetA/B/C/D/R* (12 %), followed by *bla*EC*- ant(3′) (aadA1*) (5 %), DHFR*-tetA/B/C/D/R* (3 %)*,* then *ant(3′) (aadA1)-*DHFR (2 %) (Fig. S12). Although phenotypic susceptibility tests were performed for all the isolates, phenotypic resistance to single agents was confirmed in ten isolates only: to trimethoprim in a single isolate, to sulfamethoxazole in four unrelated isolates and to tetracycline in four closely related isolates from a single animal. A single ST2076 (Achtman) isolate (PapAN-14-1) belonging to the ST349 lineage was phenotypically resistant to trimethoprim, sulfamethoxazole and tetracycline (multi-drug resistance). The associated resistance genes were harboured on an IncFIB plasmid. The genotypic resistance predictions were largely concordant with the results of phenotypic testing (range, 90–99 %, File S3). Due to logistic constraints, we could not carry out phenotypic confirmation of the predicted penicillinase-type beta-lactamase resistance.

**Fig. 5.**
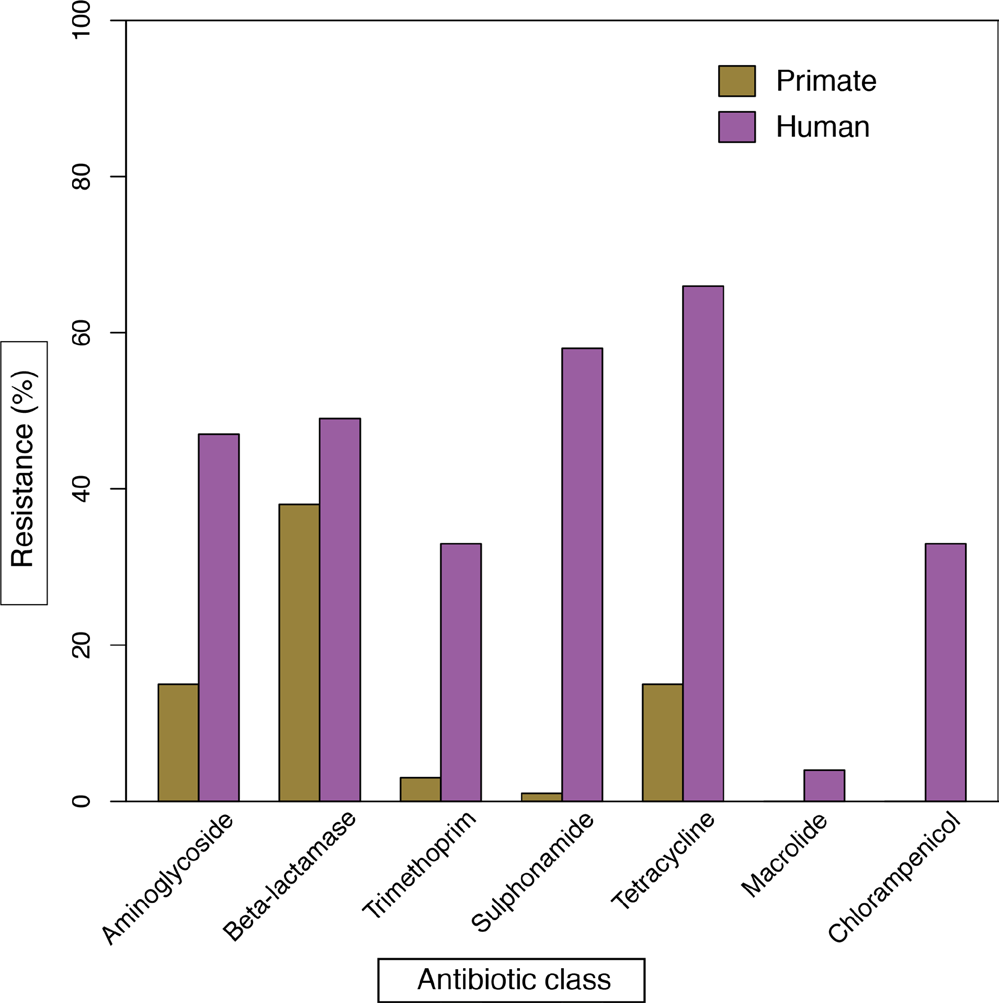
Bar graph comparing the prevalence of antimicrobial resistance genotypes in *E. coli* isolated from humans in the Gambia (*n*=128) as found in EnteroBase [30] to that found among the study isolates (*n*=101). The antimicrobial resistance genes detected were as follows: Aminoglycoside: *aph*(6)-Id, ant *aac*(3)-IIa, *ant*(3'')-Ia, *aph*(3'')-Ib, *aad*A1, *aad*A2; Beta-lactamase: *bla*EC, *bla*OXA-1, *bla*TEM-1B, *bla*TEM-1B, *bla*TEM-1C, *bla*SHV1; Trimethoprim: *dfrA/*DHFR; Sulphonamide: *sul1*, *sul2*; Tetracycline: *tet*(A), *tet*(B), *tet*(34), *tet*(D); *tet*(R) Macrolide, *mph*(A); Chloramphenicol, catA1. Screening of resistance genes was carried out using ARIBA ResFinder [38] and confirmed by ABRicate (https://github.com/tseemann/abricate). A percentage identity of ≥ 90% and coverage of ≥70% of the respective gene length were taken as a positive result.

A higher prevalence of genotypic antimicrobial resistance was found in *E. coli* isolates from humans in the Gambia, compared to what prevails in the monkey isolates (Fig. 5). Notably, a range of beta-lactamase resistance genes were found among *E. coli* from humans in the Gambia (*bla*OXA*-1, bla*TEM*-1B, bla*TEM*-1B, bla*TEM*-1C, bla*SHV*-1*), while only the *bla*EC gene occurred in our study isolates.

### Prevalence of plasmid replicons

Eighty percent (81/101) of the study isolates harboured one or more plasmids. We detected the following plasmid replicon types: IncF (various subtypes), IncB/K/O/Z, I1, IncX4, IncY, Col plasmids (various subtypes) and plasmids related to p0111 (rep B) (File S4a). Long-read sequencing of six representative samples showed that the IncFIB plasmids encoded acquired antibiotic resistance, fimbrial adhesins and colicins (File S4b). Also, the IncFIC/FII, ColRNAI, Col156 and IncB/O/K/Z plasmids encoded fimbrial proteins and colicins. Besides, the IncX and Inc-I-Aplha encoded bundle forming pili *bfp*B and the heat-stable enterotoxin protein *StbB,* respectively.

### Polished assemblies of novel strains

We generated complete genome sequences of five novel sequence types of *E. coli* (ST8525, ST8527, ST8532, ST8826, ST8827) within the seven-allele scheme (Achtman) (File S4a). Although none of these new genomes encoded AMR genes, one of them (PapRG-04–4) contained an IncFIB plasmid encoding fimbrial proteins and a cryptic ColRNA plasmid. PHASTER identified thirteen intact prophages and four incomplete phage remnants (File S4B). Two pairs of genomes from Guinea baboons from different parks shared common prophages: one pair carrying PHAGE_Entero_933W, the other PHAGE-Entero_lambda.

## Discussion

We have described the population structure of *E. coli* in diurnal non-human primates living in rural and urban habitats from the Gambia. Although our sample size was relatively small, we have recovered isolates that span the diversity previously described in humans and have also identified ten new sequence types (five of them now with complete genome sequences). This finding is significant, considering the vast number of *E. coli* genomes that have been sequenced to date (9, 597 with MLST via sanger sequencing and 127, 482 via WGS) [30].

Increasing contact between animal species facilitates the potential exchange of pathogens [55]. Accumulating data shows that ExPEC strains are frequently isolated from diseased companion animals and livestock – highlighting the potential for zoonotic as well as anthroponotic transmission [54, 56]. In a previous study, green monkeys from Bijilo Park were found to carry lineages of *Staphylococcus aureus* thought to be acquired from humans [16]. Our analyses similarly suggest exchange of *E. coli* strains between humans and wild non-human primates – with only seven cgMLST alleles separating a simian ST349 isolate and a human bloodstream isolate from Canada. This simian ST349 isolate was recovered from a green monkey in Abuko Nature Reserve, where tourists sometimes handfeed monkeys, despite prohibitions. A limitation of our study is that we could not sample *E. coli* from humans living in close proximity to the study primates. Comparisons between simian isolates and those from sympatric humans may shed light on possible transmission routes between humans and primates in this setting. However, beside human–monkey or monkey–human transmission, it is possible for the spread of pathogenic strains to have originated from an environmental reservoir to both humans and monkeys. Our results also show that non-human primates harbour *E. coli* genotypes that are clinically important in humans, such as ST73, ST127 and ST681, yet are distinct from those circulating in humans – probably reflecting lineages that have existed in this niche for long periods.

We found that several monkeys were colonized with multiple STs, often encompassing two or more phylotypes. Colonization with multiple serotypes of *E. coli* is common in humans [20–23]. Our results indicate that a single monkey can carry as many as five STs. Sampling multiple colonies from single individuals also revealed within-host diversity arising from microevolution. However, we also found evidence of acquisition in the same animal of multiple lineages of the same sequence type, although it is unclear whether this reflects a single transmission event involving more than one strain or serial transfers.

We found a relatively lower prevalence of genotypic antimicrobial resistance among our study isolates, compared to the genotypic resistance observed among isolates sourced from humans in the Gambia – probably reflecting differing selective pressures from antibiotic use. The Gambia does not have national AMR surveillance data and background data on the use of antimicrobials is limited. However, a recent study on the aetiology of diarrhoea among children less than 5 years old reported the frequent use of trimethoprim/sulphamethoxazole in the treatment of diarrhoea in the Gambia [57]. This probably accounts, at least in part, for the observed high rates of genotypic resistance to trimethoprim and sulphonamides among human *E. coli* isolates from the Gambia. The excretion of resistant bacteria and active antimicrobials from humans and domesticated animals and their persistence in the environment is known to facilitate the proliferation of AMR in the environment [19].

Antimicrobial resistance in wildlife is known to spread on plasmids through horizontal gene transfer [58]. Given the challenge of resolving large plasmids using short-read sequences [59], we exploited long-read sequencing to document the contribution of plasmids to the genomic diversity that we observed in our study population. Consistent with previous reports [60], we found IncF plasmids, which encoded antimicrobial resistance genes. Virulence-encoding plasmids, particularly colicin-encoding and the F incompatibility group ones, have long been associated with several pathotypes of *E. coli* [61]. Consistent with this, we found plasmids that contributed to the dissemination of virulence factors such as the heat-stable enterotoxin protein *StbB*, colicins and fimbrial proteins.

This study could have been enhanced by sampling human populations living near those of our non-human primates. We compensated for this limitation by leveraging the wealth of genomes in publicly available databases. Furthermore, we did not sample nocturnal monkeys due to logistic challenges; however, these have more limited contact with humans than the diurnal species. Despite these limitations, our study provides insight into the diversity and population structure of *E. coli* among non-human primates in the Gambia, highlighting the impact of human continued encroachment on natural habitats and revealing important phylogenomic relationships between strains from humans and non-human primates.

## Supplementary Data

Supplementary material 1

Supplementary material 2
