# Supplementary material for: Genomic diversity of Escherichia coli isolates from non-human primates in the Gambia
Source: Microb Genom. 2020 Sep 14;6(9):mgen000428. doi: 10.1099/mgen.0.000428 (PMC7643976; doi:10.1099/mgen.0.000428)
Supplement: Supplementary material 1 [file mgen-6-428-s001.pdf]

Genomic diversity of *Escherichia coli* isolates from non-human primates in the Gambia

Ebenezer Foster-Nyarko, Nabil-Fareed Alikhan, Anuradha Ravi, Gaëtan Thilliez, Nicholas M. Thomson, David Baker, Gemma Kay, Jennifer D. Cramer, Justin O'Grady, Martin Antonio, Mark J. Pallen

Supplementary data

Supplementary File 1: Illumina sequencing methodology
Whole-genome sequencing was carried out on the Illumina NextSeq 500 platform (Illumina, San Diego, CA). We used a modified Nextera XT DNA protocol for the library preparation as follows. The genomic DNA was normalised to 0.5 ng µl-1 with 10 mM Tris-HCl. Next, 0.9 µl of Tagment DNA buffer (Illumina Catalogue No. 15027866) was mixed with 0.09 µl of Tagment DNA enzyme (Illumina Catalogue No. 15027865) and 2.01 µl of PCR-grade water in a master-mix. Next, 3 µl of the master-mix was added to a chilled 96-well plate. To this, 2 µl of normalised DNA (1 ng total) was added, pipette-mixed and the reaction heated to 55°C for 10 min on a PCR block. To each well, we added 11 µl of KAPA2G Robust PCR master-mix (Sigma Catalogue No. KK5005), comprising 4 µl KAPA2G buffer, 0.4 µl dNTPs, 0.08 µl polymerase and 6.52 µl PCR-grade water, contained in the kit per sample. Next, 2 µl each of P7 and P5 Nextera XT Index Kit v2 index primers (Illumina Catalogue numbers FC-131-2001 to 2004) were added to each well. Finally, the 5 µl of Tagmentation mix was added and mixed. The PCR was run as follows: 72°C for 3 min, 95°C for 1 min, 14 cycles of 95°C for 10 sec, 55°C for 20 sec and 72°C for 3 min. Following the PCR, the libraries were quantified using the Quant-iT dsDNA Assay Kit, high sensitivity kit (Catalogue No. 10164582) and run on a FLUOstar Optima plate reader. After quantification, libraries were pooled in equal quantities. The final pool was double-SPRI size-selected between 0.5 and 0.7x bead volumes using KAPA Pure Beads (Roche Catalogue No. 07983298001). We then quantified the final pool on a Qubit 3.0 instrument (Invitrogen, MA, USA) and ran it on a high sensitivity D1000 ScreenTape (Agilent Catalogue No. 5067-5579) using the Agilent TapeStation 4200 to calculate the final library pool molarity. The pooled library was run at a final concentration of 1.8 pM on an Illumina NextSeq500 instrument using a mid-output flow cell (NSQ® 500 Mid Output KT v2 300 cycles; Illumina Catalogue No. FC-404-2003) following the Illumina recommended denaturation and loading parameters, which included a 1% PhiX spike (PhiX Control v3; Illumina Catalogue FC-110-3001). The data was uploaded to BaseSpace (http://www.basespace.illumina.com) and then converted to FASTQ files.

Supplementary figures

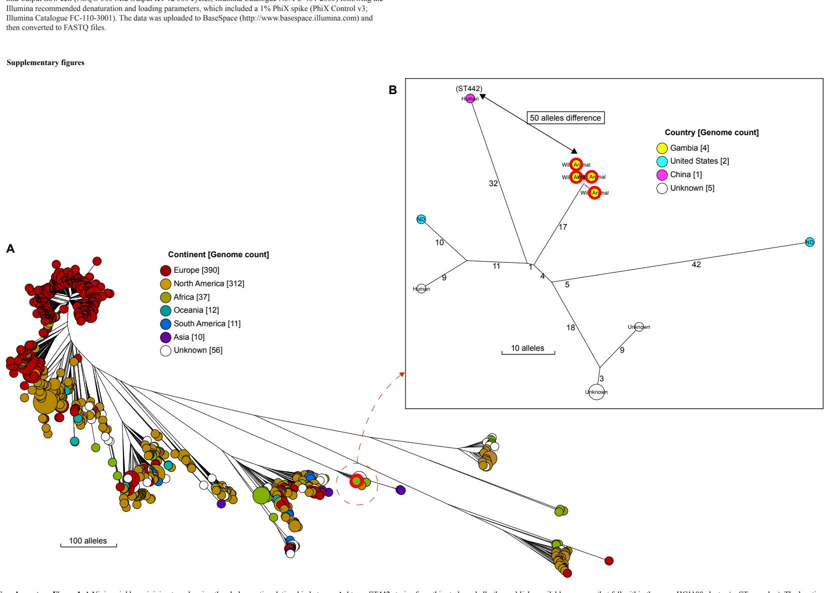

Supplementary Figure 1. A Ninja neighbour-joining tree showing the phylogenetic relationship between Achtman ST442 strains from this study and all other publicly available genomes that fell within the same HC1100 cluster (cgST complex). The locations of the isolates are displayed, with the genome count displayed in parenthesis. Branch lengths display the allelic distances separating genomes. Gambian strains are highlighted in red. The sub-tree (B) shows the closest relatives to the study strains, with the allelic distance separating them displayed with the arrow. Dotted lines represent long branches which have been shortened.

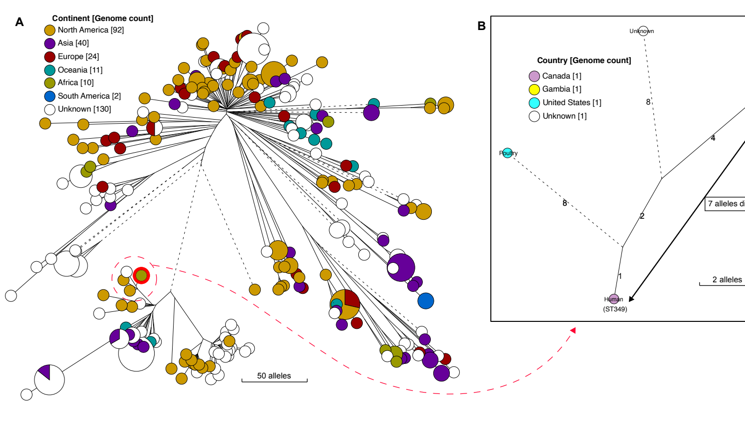

Supplementary Figure 2. A Ninja neighbour-joining tree showing the phylogenetic relationship between the ST349 (Achtman) strain from this study and all other publicly available genomes within the same HC1100 cluster (cgST complex). The legend shows the locations of the isolates, with genome counts displayed in parenthesis. Gambian strains are highlighted in red. The study ST349 strain is separated from a clinical ST349 strain by only seven alleles (<7 SNPs), as depicted in the subtree (B). Long branches are shortened (indicated by dashes).

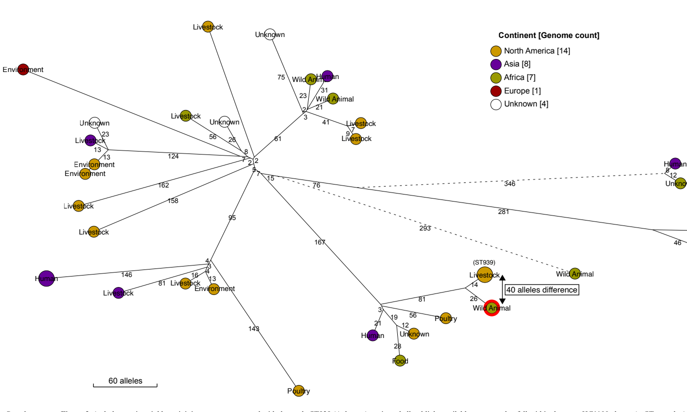

Supplementary Figure 3. A phylogenetic neighbour-joining tree reconstructed with the study ST939 (Achtman) strain and all publicly available genomes that fell within the same HC1100 cluster (cgST complex). The legend shows the locations of the isolates, with red highlights around the nodes indicating the Gambian strains. The allelic distance between the study strain and its nearest relative, a bovine ST939 strain, has been given, depicted by the arrow. Dotted lines indicate shortened long branches.

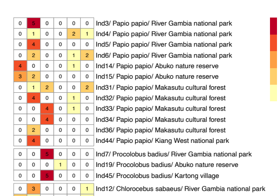

Supplementary Figure 4. Distribution of phylotype per individual. The numbers depict the frequency of the respective phylotype (A, B1, B2, D, E and Clade I) per the study monkey from whom they were isolated (depicted on the y-axis). Ind, individual.

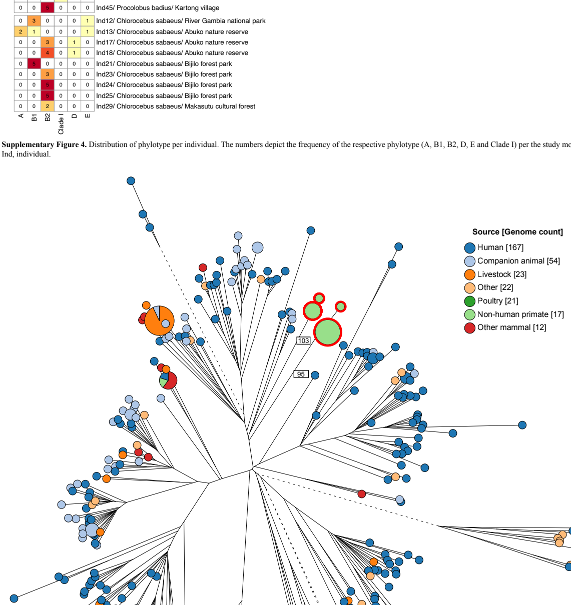

Supplementary Figure 5. A Ninja neighbour-joining tree reconstructed with Achtman ST73 colibactin+ strains from this study and all other publicly available ST73 (Achtman) strains that fell within the same HC1100 cluster (cgST complex) in EnteroBase (Reference 41). The sources of the isolates are displayed, with Gambian strains highlighted in red. The Gambian non-human primate strains are on separate long branches, although nested within clades populated by human strains from other countries, suggestive of probably an ancient transmission between the two hosts. The branch lengths for the Gambian strains are displayed. Dotted lines represent long branches which have been shortened.

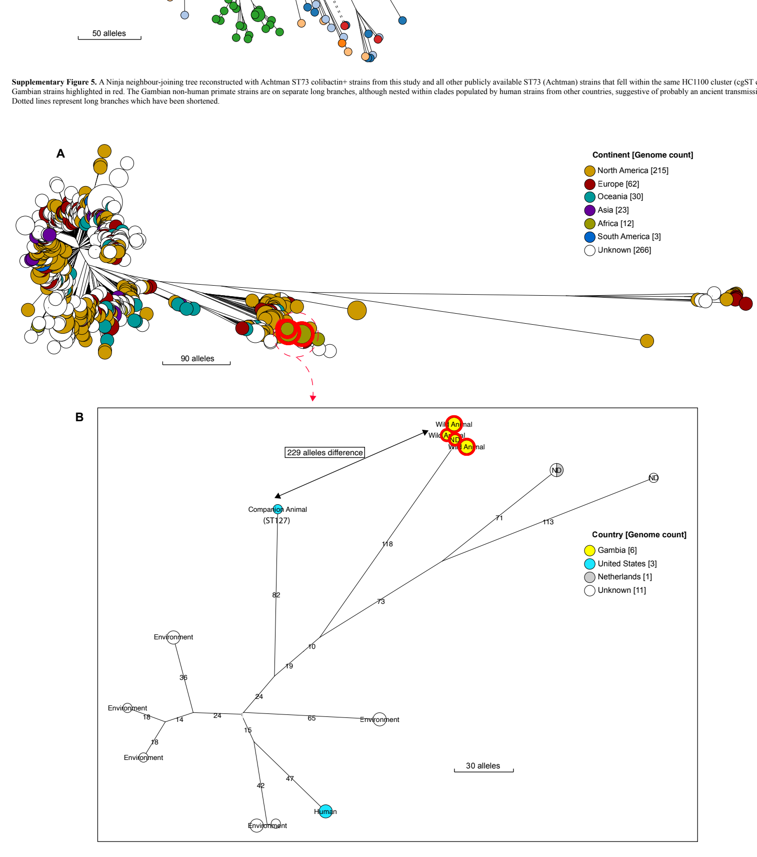

Supplementary Figure 6. A Ninja neighbour-joining tree showing the phylogenetic relationship between ST127 strains from this study and other publicly available strains that occur within the same HC1100 cluster (cgST complex). The sources of the isolates are displayed in the legends, with Gambian strains highlighted in red. Branch lengths display the allelic distances separating genomes. The sub-tree (B) shows the closest relatives to the study strains, with the allelic distances separating them displayed with the arrow. Dotted lines represent long branches which have been shortened. Dotted lines represent long branches which have been shortened.

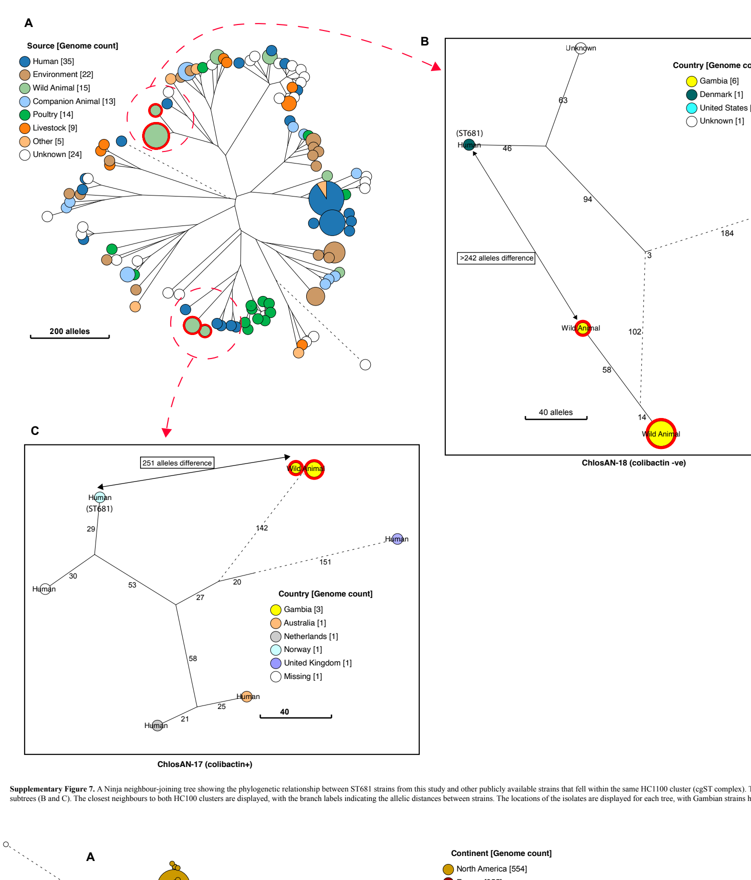

Supplementary Figure 7. A Ninja neighbour-joining tree showing the phylogenetic relationship between ST681 strains from this study and other publicly available strains that fell within the same HC1100 cluster (cgST complex). The study strains fell into two separate HC100 clusters, which are depicted in the two subtrees (B and C). The closest neighbours to both HC100 clusters are displayed, with the branch labels indicating the allelic distances between strains. The locations of the isolates are displayed for each tree, with Gambian strains highlighted in red. Dotted lines represent long branches which have been shortened.

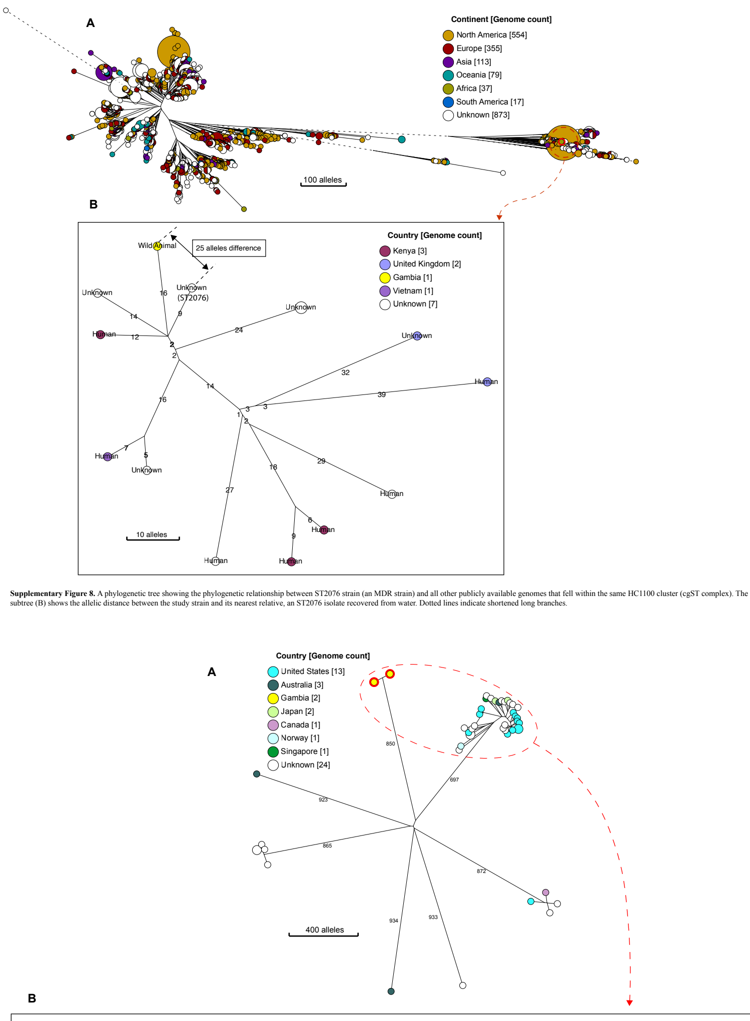

Supplementary Figure 8. A phylogenetic tree showing the phylogenetic relationship between ST2076 strain (an MDR strain) and all other publicly available genomes that fell within the same HC1100 cluster (cgST complex). The legend shows the locations of the isolates, Gambian strains are highlighted in red. The subtree (B) shows the allelic distance between the study strain and its nearest relative, an ST2076 isolate recovered from water. Dotted lines indicate shortened long branches.

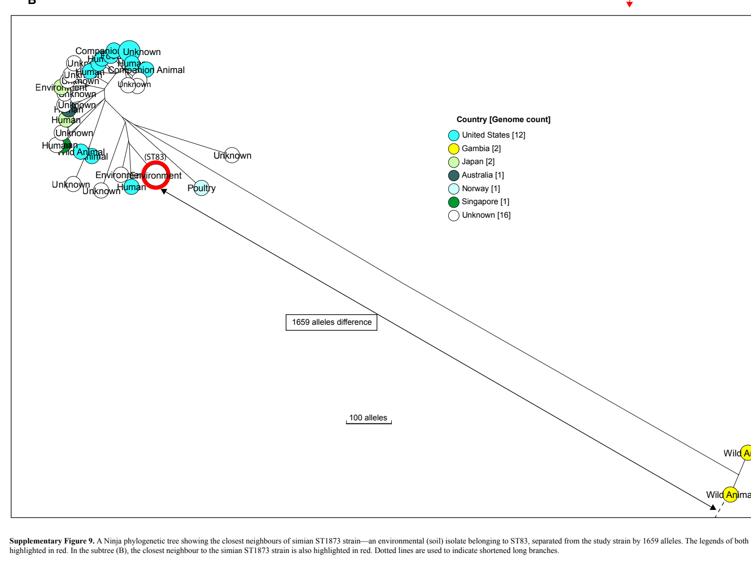

Supplementary Figure 9. A Ninja phylogenetic tree showing the closest neighbours of simian ST1873 strain—an environmental (soil) isolate belonging to ST83, separated from the study strain by 1659 alleles. The legends of both the main tree and the subtree show the locations of the isolates Gambian strains are highlighted in red. In the subtree (B), the closest neighbour to the simian ST1873 strain is also highlighted in red. Dotted lines are used to indicate shortened long branches.

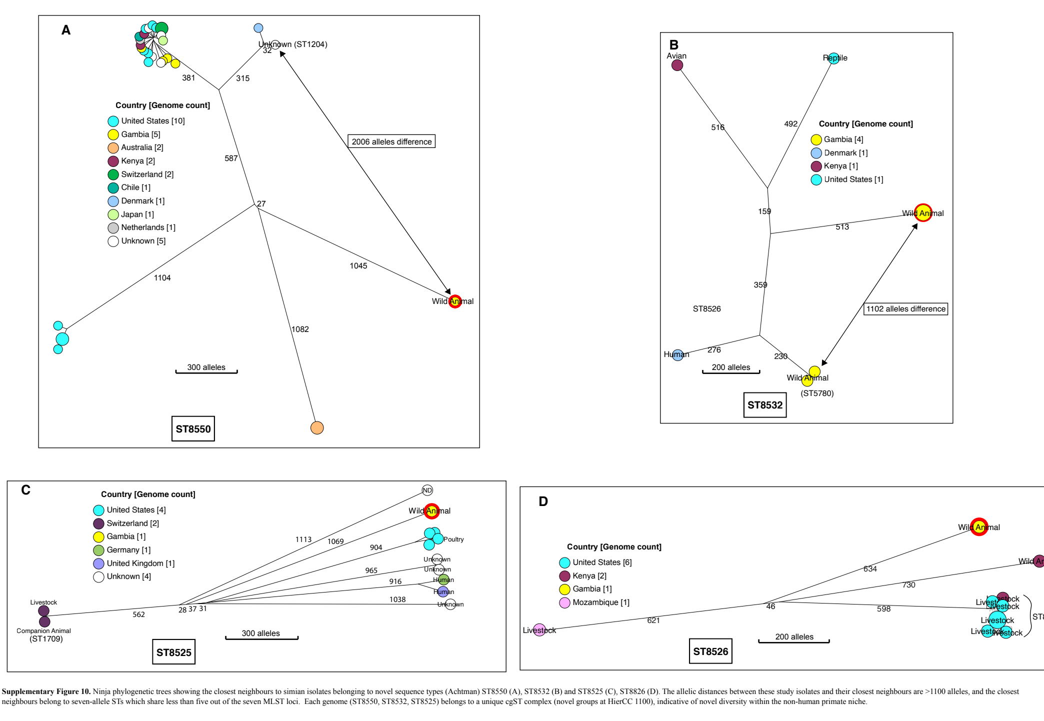

Supplementary Figure 10. Ninja phylogenetic trees showing the closest neighbours to simian isolates belonging to novel sequence types (Achtman) ST8550 (A), ST8532 (B) and ST8525 (C), ST8826 (D). The allelic distances between these study isolates and their closest neighbours are >1100 alleles, and the closest neighbours belong to seven-allele STs which share less than five out of the seven MLST loci. Each genome (ST8550, ST8532, ST8525) belongs to a unique cgST complex (novel groups at HierCC 1100), indicative of novel diversity within the non-human primate niche.

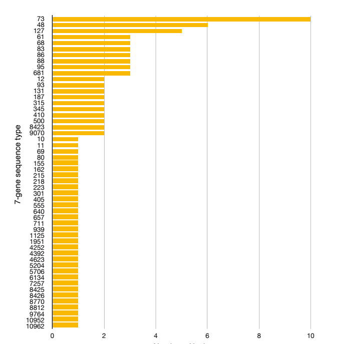

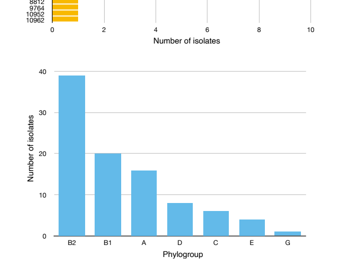

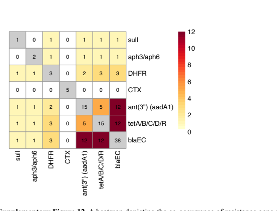

Supplementary Figure 12. A heatmap depicting the co-occurrence of resistance gene networks among the study isolates. The diagonal numbers (grey shading) shows the number of isolates carrying a particular gene, while the intersection shows the number of isolates harbouring a pair of resistance genes.
